# Molecular markers for artemisinin and partner drug resistance in natural *Plasmodium falciparum* populations following increased insecticide treated net coverage along the slope of mount Cameroon: cross-sectional study

**DOI:** 10.1186/s40249-017-0350-y

**Published:** 2017-11-06

**Authors:** Tobias O. Apinjoh, Regina N. Mugri, Olivo Miotto, Hanesh F. Chi, Rolland B. Tata, Judith K. Anchang-Kimbi, Eleanor M. Fon, Delphine A. Tangoh, Robert V. Nyingchu, Christopher Jacob, Roberto Amato, Abdoulaye Djimde, Dominic Kwiatkowski, Eric A. Achidi, Alfred Amambua-Ngwa

**Affiliations:** 1 Department of Biochemistry and Molecular Biology, University of Buea, Buea, Cameroon; 2 Department of Microbiology and Parasitology, University of Buea, Buea, Cameroon; 3 Mahidol-Oxford Research Unit, Faculty of Tropical Medicine, Mahidol University, Bangkok, Thailand; 4 Department of Zoology and Animal Physiology, University of Buea, Buea, Cameroon; 5 Department of Medical Laboratory Science, University of Buea, Buea, Cameroon; 6 Wellcome Trust Sanger Institute, Hinxton, Cambridge, CB10 1SA UK; 7 Malaria Research and Training Centre, Bamako, Mali; 8 MRC Unit, Fajara, The Gambia

**Keywords:** Molecular markers, Drug resistance, *Plasmodium falciparum*

## Abstract

**Background:**

Drug resistance is one of the greatest challenges of malaria control programmes, with the monitoring of parasite resistance to artemisinins or to Artemisinin Combination Therapy (ACT) partner drugs critical to elimination efforts. Markers of resistance to a wide panel of antimalarials were assessed in natural parasite populations from southwestern Cameroon.

**Methods:**

Individuals with asymptomatic parasitaemia or uncomplicated malaria were enrolled through cross-sectional surveys from May 2013 to March 2014 along the slope of mount Cameroon. *Plasmodium falciparum* malaria parasitaemic blood, screened by light microscopy, was depleted of leucocytes using CF11 cellulose columns and the parasite genotype ascertained by sequencing on the Illumina HiSeq platform.

**Results:**

A total of 259 participants were enrolled in this study from three different altitudes. While some alleles associated with drug resistance in *pfdhfr, pfmdr1 and pfcrt* were highly prevalent, less than 3% of all samples carried mutations in the *pfkelch13* gene, none of which were amongst those associated with slow artemisinin parasite clearance rates in Southeast Asia. The most prevalent haplotypes were triple mutants *Pfdhfr* **I**_51_ **R**_59_ **N**_108_ **I**_164_(99%), *pfcrt-* C_72_V_73_ **I**_**74**_ **E**_**75**_ **T**_**76**_ (47.3%), and single mutants *Pfdhps*S_436_ **G**_437_K_540_A_581_A_613_(69%) and *Pfmdr1* N_86_ **F**_184_D_1246_ (53.2%).

**Conclusions:**

The predominance of the *Pf pfcrt* CV**IET and** *Pf* *dhfr* **IRN** triple mutant parasites and absence of *pfkelch13* resistance alleles suggest that the amodiaquine and pyrimethamine components of AS-AQ and SP may no longer be effective in their role while chloroquine resistance still persists in southwestern Cameroon.

**Electronic supplementary material:**

The online version of this article (doi:10.1186/s40249-017-0350-y) contains supplementary material, which is available to authorized users.

## Multilingual abstracts

Please see Additional file 1 for translations of the abstract into the five official working languages of the United Nations.

## Background

Malaria is still a leading cause of illness and death especially in sub-Saharan African children under the age of five [1]. Case management currently relies largely on the use of a few effective antimalarials and is being compromised by the development and spread of resistance [2]. Parasite resistance to antimalarial drugs represents a major obstacle to malaria containment efforts [1, 3, 4]. Indeed the policy change to artemisinin-based combination therapies (ACT) for treatment of uncomplicated malaria [5], was due to the emergence and spread of resistance to chloroquine (CQ), sulphadoxine-pyrimethamine (SP) and other monotherapies [6, 7]. However, the emergence of artemisinin resistance in Western Cambodia of Southeast Asia (SEA) [1, 3] has prompted global concern given that CQ and SP resistance arose in the same region and then spread to Sub-Saharan Africa (SSA) [8]. Recent studies also suggest that resistant mutations may emerge independently in SEA and SSA [9–13] necessitating regional molecular monitoring of markers for the control and containment of resistant parasites. Information on parasite resistance to artemisinins, ACT partner drugs or to previously withdrawn antimalarials is vital for malaria control [14] and could justify the re-introduction of abandoned drugs [15] since drug-sensitive populations of *Plasmodium falciparum* resurge following long-term drug withdrawal.

Single nucleotide polymorphisms (SNPs) have been fundamental in monitoring existing or predicting emerging drug resistance patterns. Chloroquine resistance is linked to mutations in the *P. falciparum* chloroquine resistance transporter (*Pfcrt*) [16–18], and is associated with mutations in codons 72–76. The *Pfcrt* Lysine to Threonine substitution at position 76 (K76T) [16] is considered to be critical to CQ resistance as well as to the structurally and similarly acting drug, amodiaquine (AQ) [19]. SNPs in the *P. falciparum* multidrug resistance 1 (*Pfmdr1)* gene, notably the *Pfmdr1* N86Y substitution [20], have been associated with resistance to CQ [21], mefloquine, halofantrine, and quinine [22]. Artemether lumefantrine (AL), the most commonly used ACT in SSA [1] seems to select *pfcrt* and *pfmdr1* SNPs in parasite reinfections [23, 24], with a high proportion of *pfmdr1 -* N86 alleles recorded in AL treated patients with recurrent parasites [25]. SP resistance is due to point mutations in the parasite dihydrofolate reductase (*dhfr*) and dihydpteroate synthetase (*dhps*) genes that confer resistance to pyrimethamine and sulphadoxine respectively [26, 27].

Mutations in *P. falciparum Kelch13* have been shown to underlie artemisinin resistance [13, 28], with nonsynonymous polymorphisms in the propeller domain validated as molecular markers for determining the emergence and spread of artemisinin-resistant *P. falciparum* [28, 29]. While the four core mutations have not been detected in Africa, several other non-synonymous K13 mutations have been identified and the effect of these and markers of previous antimalarial resistance remains largely unknown. The A481V and G533C substitutions, for instance, have been confirmed to be adjacent to these four major SNPs and may affect the tertiary structure and thus the function of the propeller [29, 30].

This ever evolving parasite population dynamics necessitates antimalarial resistance monitoring in distinct transmission contexts. Although drug pressure is the primary driver of anti-malarial drug resistance, alterations in malaria transmission has also been implicated [31]. In areas where drug policy has changed and the insecticide treated net coverage has been scaled up, molecular monitoring of current and previously used drugs could provide a better understanding of the impact of these factors on drug resistance alleles [6]. In Cameroon, CQ, AQ and SP were administered as monotherapies during 1999–2004, with CQ used as first line drug for treatment of malaria until 2002, when an interim policy was adopted involving the use of AQ as the alternative first line drug for uncomplicated malaria while SP was the second line drug [7]. Due to the declining efficacy of *P. falciparum* to AQ and SP, the Cameroon Ministry of Public Health revised its treatment policy in 2004 to artemisinin-based combination therapy (ACT) and adopted AS-AQ as first line drug for uncomplicated malaria while quinine (QN), injectable Arthemeter (or QN) and SP were recommended for *P. falciparum* treatment failure, severe malaria and intermittent preventive treatment of malaria in pregnancy (IPTp), respectively [1]. A number of other ACT options are available for treatment of mild malaria in Cameroon [32], with artemether-lumefantrine (AL) reportedly prescribed by up to 36.6% of health workers in a recent study [33].

The government of Cameroon embarked on a scaling-up of ITN coverage in 2011, in line with the Roll Back Malaria recommendation of universal coverage [34]. In the study area, where malaria parasitaemia is higher in the rainy seasons [35] and at lower altitude [36], significant increases in ITN ownership and usage have been reported [37]. This, together with infrastructural development in the area may have altered the structure of the vector population, transmission of infection, genetic diversity of circulating parasites and the efficacy of antimalarials. However, other factors such as host immunity may also be important determinants of treatment failure and the emergence and transmission potential of resistant parasites [38–40].

Reports on the monitoring of antimalarial resistance markers in Cameroon have been limited manly to the *pfcrt:* K76T [41–44] and *pfmdr1:* N86Y [42, 44]. The *pfcrt*: K76, for instance, has remained relatively fixed at 12% in 2000 [41] compared to 13% in 2012 [45]. The only such study in the mount Cameroon area [44] revealed that 87% and 76% of samples between 2004 and 2006 carried the *pfcrt:* K76T and *pfmdr1:* N86Y alleles, respectively. Furthermore, there have been no reports on the prevalence of molecular markers of artemisinin resistance in the area. In this study, the prevalence of mutations in genes associated with drug resistance were assessed in natural parasite populations across different altitudinal zones from southwestern Cameroon, enriching data on parasite antimalarial resistance, with implications for the control of the disease.

## Methods

### Study area

The study was conducted in localities on the eastern slope of Mt. Cameroon, with varying altitudes as described [37]. The area is categorized by an equatorial climate comprising two seasons: a short dry season (November–March) and a long rainy season (March–November) [35], intense and perennial *Plasmodium* spp. transmission and higher parasite prevalence in the rainy season and at lower altitude [36, 46]. *P. falciparum* is responsible for most of the malaria infections [1] while *Anopheles gambiae* (*Anopheles coluzzii* M form) is the main malaria vector species, with overall Entomological Inoculation Rates (EIR) as high as 287 infective bites/person/year [35]. There is a substantial level of human migration between localities, mainly for educational, recreational and commercial purposes.

### Study design and selection of sampling sites and participants

This was a cross-sectional community - and hospital – based study conducted between May 2013 and March 2014. Individuals with asymptomatic parasitaemia (AP) were enrolled through surveys from selected rural and semi-urban communities at varying altitudes as described [37] based on previous reports of variation in malaria parasitaemia [38, 46]. Three communities, Mutengene, Ombe and Tiko below 200 m were considered to be at low altitude while Mile 14, 15, 16, Muea and Molyko located between 385 and 575 m were considered to be at intermediate altitude. Individuals residing from checkpoint to Buea Town and Tole above 636 m were considered to be at high altitude. Uncomplicated Malaria (UM) subjects were also registered from health facilities within these communities. All local residents, with a minimum of 1000 asexual parasites per microliter of peripheral blood, who had not travelled out of the target sites within the last 3 weeks were eligible for enrollment. A structured questionnaire was used to record demographic and clinical data such as age, area of residence and drug history of all participants. All patients were given oral antimalarial, based on their weight, by the attending clinician and according to national guidelines.

### Sample collection and parasite detection

Prospective participants were prescreened by light microscopy using giemsa-stained thick and thin blood smears of the peripheral blood as described previously [37]. A smear was only considered negative if no malaria parasites were seen in 100 high power fields. The level of parasitaemia in positive smears was estimated by counting the parasites against a minimum of 200 white blood cells and assuming a leucocyte count of 8000 per microliter of blood [36, 47]. Quality control was ensured in accordance with the World Health Organisation’s protocol [47]. Venous blood (3–5 ml) was then collected from *P. falciparum* positive participants into EDTA tubes for molecular analysis.

### DNA extraction

Leucocytes were depleted from whole blood using CF11 cellulose columns (4021–050) following a modified WorldWide Antimalarial Resistance Network (WWARN) MOL02 protocol (www.wwarn.org). Parasite genomic DNA was then extracted using a commercial kit (Qiagen, UK) according to manufacturer’s instructions, eluted with 100 μl TE (10 mM Tris–HCl; 0.5 mM EDTA; pH 9.0) elution buffer (Qiagen, UK) and kept at −34 °C until genotyping.

### Genotyping of mutations in drug resistance genes

Samples with >50 ng DNA and <80% human DNA contamination (239/259, 92.3%) were sequenced on the Illumina HiSeq platform (Illumina, San Diego, USA), and subsequently genotyped using well established methods, as described previously [13, 48] without any modification. In brief, samples were genotyped at each SNP on the basis of sequencing read counts, with at least 5 reads required to emit a genotype and at least 2 reads to call an allele. *Pfkelch13* alleles were determined by identifying any variation across the gene that would result in a non-synonymous change in the protein, as described [49].

Haplotypes were constructed independently for each locus. As it is impossible to ascertain if any two haplotypes are coming from the same genome for complexity of infection (COI) > 1, only the frequency of haplotypes without any heterozygous call were reported. The sample should therefore carry the same DR haplotype even if multiple genomes are present in the infections.

### Complexity of infection

Complexity of Infection was determined using the program COIL [50]. From the MalariaGEN Plasmodium falciparum Community Project data resource (https://www.malariagen.net/projects/p-falciparum-community-project), 101 genomic SNPs of mid-high MAF with large between-population Fst were used as a “barcode” within COIL to estimate COI. COIL was used with default parameters and population allele frequency estimate were calculated from sample data, not pre-determined.

### Statistical analyses

All data were entered into Excel and analyzed using SPSS Statistics 20 for windows (SPSS Inc., Chicago, USA). The significance of difference in prevalence were explored using the Pearson’s chi square test whereas the differences in group means were assessed using Student’s *t* – test or analyses of variance (ANOVA). A difference giving a *P* value ≤0.05 was considered statistically significant.

## Results

### Characteristics of smear-positive participants

A total of 259 participants were enrolled in this study from three different altitudes (Table 1), most of whom had uncomplicated malaria (74.8%, 190/254), reportedly had fever in the previous 48 h (67.5%), were anaemic (47.2%) and females (52.1%). The mean age (± SD), geometric mean parasite density and complexity of infection (± SD) were 13.9 ± 13.09 years, 15,715 parasites/μl blood and 1.81 ± 1.10 respectively. The proportion of individuals with asymptomatic parasitaemia from the community surveys at low, intermediate and high altitude was 6.0%, (11/184), 7% (34/487) and 14.7% (19/129) respectively.

**Table 1:** Basic characteristics of *Plasmodium falciparum* smear-positive participants at different altitudes along the slope of mount Cameroon, southwestern Cameroon

Characteristic	All participants		MT <200 masl	MM 385–575 masl	CB ≥ 626 masl	*P*-value
	*n*	Values				
Age (mean ± SD) [Range] / years	246	13.6 ± 12.8 [0.5–65]	7.7 ± 8.6 (32)	14.1 ± 11.4 (160)	15.6 ± 17.1 (54)	*0.014*
Weight (mean ± SD) [Range] / kg	217	37.2 ± 24.7 [2.0–110.0]	22.6 ± 13.7 (30)	40.1 ± 24.5 (137)	38.2 ± 27.5 (50)	*0.002*
Male: Female ratio	246	47.9: 52.1	40.6: 59.4 (32)	49.7: 50.3 (159)	47.3: 52.7 (55)	0.641
Clinical phenotype (AP: UM)	244	25.2: 74.8	35.5: 64.5 (31)	21.5: 78.5 (158)	34.5: 65.5 (55)	0.076
GMPD [Range] (parasites/μl blood)	246	15,715 [1267–1,840,000]	22,387 (32)	16,254 (160)	11,216 (54)	0.081
COI (mean ± SD) [Range]	230	1.8 ± 1.1 [1–5]	2.1 ± 1.2 (29)	1.8 ± 1.1 (147)	1.7 ± 1.0 (54)	0.222
Hb (mean ± SD) [Range] / g/dl	235	10.7 ± 2.1 [6.0–19.0]	10.2 ± 2.3 (30)	10.8 ± 2.0 (154)	10.5 ± 2.1 (51)	0.254
Anaemic [% (*n*)] (Hb < 11 g/dl)	235	47.2 (111)	53.3 (30)	42.2 (154)	58.8 (51)	0.093
Fever in last 48 h [% (*n*)]	235	67.5 (155)	64.5 (31)	84.7 (150)	72.7 (55)	*0.017*

Values in italics depeict significant *p* values for differences in group means or proportions; *AP* Asymptomatic parasitaemia, *COI* Complexity of infection, *UM* Uncomplicated malaria, *GMPD* Geometric mean parasite density, *masl* altitude (in metres) above sea level, *MT* Mutengene & Tiko, *MM* Mile 14 to Muea transect, *CB* Checkpoint-Molyko to Buea Town transect

### Prevalence of drug resistance molecular markers

**Fig. 1:**
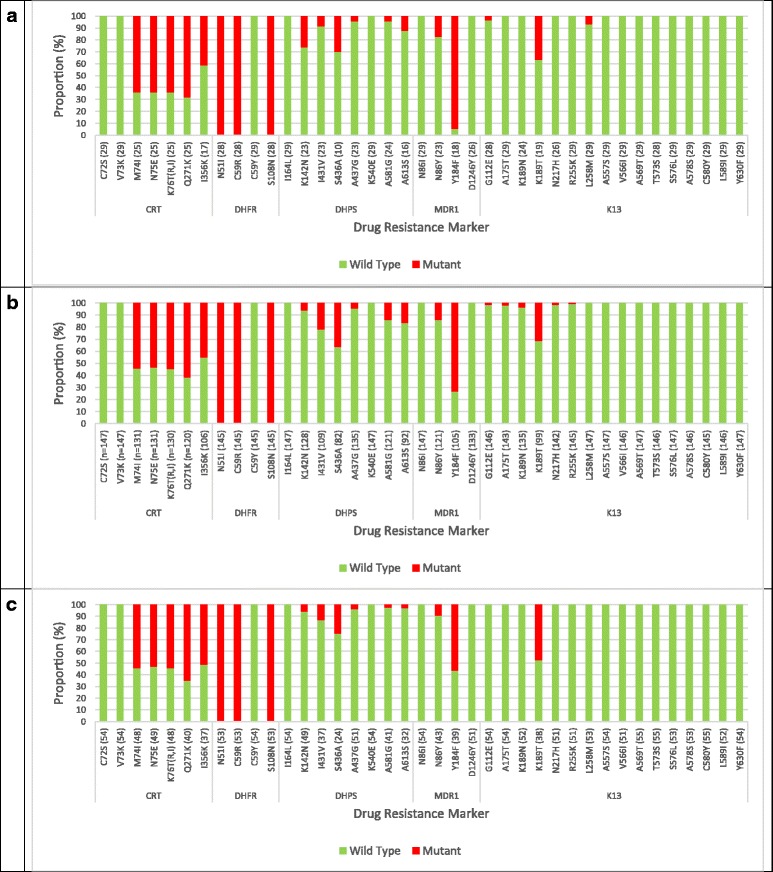
Prevalence of mutation in different marker genes in natural *Plasmodium falciparum* populations along the slope of mount Cameroon (**a** = low altitude (MT); (**b**) = Intermediate altitude (MM); (**c**) = High altitude (CB); numbers in brackets after marker name indicate samples effectively genotyped)

Some resistance mutations were highly prevalent; all 233 (100%) samples had *pfdhfr*: S108N and 232 (more than 99%) had *pfdhfr*: N51I and C59R mutant alleles (Table 2). One hundred and twenty one (72%) of samples harbored mutations at *pfmdr1*: Y184F while 120 (62.5%) mutations were detected in *pfcrt: Q*271K overall. CQ resistance alleles were also prevalent, with at least 115 (50%) parasites carrying mutations in *pfcrt* at codons 74, 75, and 76. However, 5 (less than 3% of all samples) had *pfkelch13* (*pk13*) mutant alleles, except for *pfk13*: 189 T detected in 58 (36%) samples overall. Furthermore, none of the samples carried mutations in *pfk13* at codons 112, 175, 217, 255, 258, 569, 573, 578 and 580, *pfcrt* at codon 72, *pfdhfr* at codons 59 and 164, *pfdhps* at codon 540 and *pfmdr1* at codon 86 (Fig. 1).

**Table 2:** Most prevalent drug resistant mutations in *P. falciparum* isolates from the slope of mount Cameroon

Gene	Codons	This study Prevalence (isolates)	Previous reports in area^α^ or elsewhere in Cameroon^β^		
			Prevalence (isolates)	Year of sampling	Reference
*Pfcrt*	M74I	55.0 (116)	NR	NR	NR
	N75E	54.2 (115)	NR	NR	NR
	K76 T(R,I)	55.2 (116)	87.1	2004–2006	Mbacham et al., 2010^α^ [44]
	Q271K	62.5 (120)	NR	NR	NR
	I356K	46.1 (77)	NR	NR	NR
*Pfdhfr*	N51I	99.6 (232)	96.1 (51)	2010–2011	Chauvin et al., 2015^β^ [63]
	C59R	99.6 (232)	98 (51)		
	S108 N	100 (233)	98 (51)		
*Pfdhps*	K142 N	9.2 (19)	NR	NR	NR
	I431V	17.6 (31)	9.8 (51)	2010–2011	Chauvin et al., 2015^β^ [63]
	S436A	32.0 (39)	47.1 (51)		
	A581G	9.8 (19)	5.9 (51)		
	A613S	12.3 (18)	11.8 (51)		
*Pfmdr1*	N86Y	13.0 (25)	73.8	2004–2006	Mbacham et al., 2010^α^ [44]
	Y184F	72.0 (121)	9 (64)	1997–2000	Basco et al., 2002^β^ [41]
*Pfkelch13*	K189 T	36.0 (58)	NR	NR	NR

α and β denote reports from the study area and elsewhere in Cameroon respectively; NR = No Reports

The prevalence of *pfdhps*: K142N (*P* = 0.006) and *pfmdr1*: Y184F (*P* = 0.010) mutations, but not other markers, differed significantly among study sites, highest in the Mutengene – Tiko area (Fig. 1, Additional file 2: Table S1). However, the proportion of the alleles was similar between AP and UM individuals in all study sites, except for *dhps*: I431V that was higher (*P* = 0.039) in UM (22/86, 25.6%) compared to AP (1/22, 4.5%) at MM. Two hundred and thirty two (99.6%) samples harbored the *dhfr* N51I/C59R/S108 N (IRN) triple mutant, while none had the *dhps* A437G/K540E (GE) double mutant and therefore the IRN + GE quintuple mutant haplotype.

### Pfk13 mutations

None of the candidate and validated non-synonymous *K13* resistance mutations were detected in the 239 samples analyzed (Fig. 1). Furthermore, other less frequent variants that have been associated with in vivo or in vitro tests, or both were not seen in all samples analyzed.

### Haplotypes of CQ and SP markers

#### CQ resistance haplotypes

The prevalence of *pfcrt* and *pfmdr1* haplotypes in the study area are shown (Table 3). Two different *pfcrt* haplotypes were observed, with 60 (25.3%) of the samples bearing wild type alleles at all five codons, C_72_V_73_M_74_N_75_K_76_ (CVMNK) while the majority (112, 47.3%) carried triple mutations at codons 74, 75, and 76 (CV**IET**). Nevertheless, 65 (27.4%) had a mixture of the CV**IET** and CVMNK haplotypes. In all, 177 (74.7%) of the samples had the CV**IET** haplotype, which was more prevalent (*P* = 0.011) in semi-urban settings (50.2%) compared to rural settings (31.2%) (Table 3). The alternative South American-type mutant haplotype (SVMNT) was not detected.

**Table 3:** Prevalence of point mutation haplotype in the *P. falciparum* CQ resistance transporter, dihydrofolate reductase, dihydropteroate synthetase, and multidrug resistance 1 genotypes among clinical samples from different localities in south western Cameroon

Drug implicated	Gene (codons)	Haplotype (amino acids)	Prevalence [*n* (%)]	Area (%)			*P*-value
				MT	MM	CB	
CQ, AQ, LM	*pfcrt* (72–76)	CV**IET**	112 (47.3)	51.7	46.6	47.3	0.905
		CVMNK	60 (25.3)	27.6	23.6	25.5	
		CV**IET** + CVMNK	65 (27.4)	20.7	29.7	27.3	
SP	*pfdhfr* (51, 59, 108, 164)	**IRN**I	232 (99.6)	100	99.3	100	0.754
		NC**N**I	1 (0.4)	0.0	0.7	0.0	
	*pfdhps* (436, 437, 540, 581, 613)	S**G**KAA	67 (69.1)	66.7	65.1	75.0	0.925
		**AG**KAA	10 (10.3)	22.2	7.9	15.0	
		**AG**K**GS**	10 (10.3)	11.1	12.7	5.0	
		**A**AKAA	5 (5.2)	0.0	6.3	5.0	
		**AG**KA**S**	3 (3.1)	0.0	4.8	0.0	
		**A**AK**GS**	1 (1.0)	0.0	1.6	0.0	
		SAKAA	1 (1.0)	0.0	1.6	3.0	
AQ, CQ, LM, MQ	*pfmdr-1 (86, 184, 1246)*	N**F**D	74 (53.2)	71.4	55.7	40.0	0.169
		NYD	46 (33.1)	7.1	30.7	48.6	
		**YF**D	18 (12.9)	21.4	12.5	11.4	
		**Y**YD	1 (0.7)	0.0	1.1	0.0	

Boldface letters depict mutant alleles; *AQ* amodiaquine, *CQ* chloroquine, *LM* lumefantrine, *MQ* mefloquine, *SP* sulphadoxine–pyrimethamine, *MT* Mutengene & Tiko, *MM* Mile 14 to Muea transect, *CB* Checkpoint-Molyko to Buea Town transect

A total of four *pfmdr1* haplotypes were detected in the area, with 46 (33.1%) samples containing wild type alleles at codons 86, 184 and 1246, *pfmdr1* N_86_ **Y**_184_D_1246_ (NYD). Nevertheless, the predominant haplotype (74, 53.2%), contained a single mutation at codon 184 (Y**F**D) whereas 18 (12.9%) and 1 (0.7%) of samples had haplotype variants with double (**YF**D) and single (**Y**YD) mutations respectively. The proportion of the *pfmdr1* haplotypes did not vary with locality (Table 3).

#### SP resistance haplotypes

A total of nine distinct haplotypes were detected in the study area, with the proportion of the different variants independent of locality of residence (Table 2). At least 232 (99%) and 67 (69%) of samples in all localities harbored the *pfdhfr* N_51_I/C_59_R/S_108_N/I_164_ (IRNI) and *pfdhps* S_436_/A_437_G/K_540_/A_581_/A_613_ (SGKAA) haplotypes respectively. Overall, 73.3% and 4.4% of isolates in the area harbored the **IRN**I + S**G**KAA and **IRN**I + **A**AKAA haplotypes carrying quadruple mutations at the key *dhfr* and *dhps* codons respectively. Furthermore, 11 and 3 samples had the sextuple mutant **IRN**I + **A**AK**GS** and **IRN**I + **AG**KA**S** respectively while 7.4% and 3.0% had the quintuple mutant **IRN**I + **AG**KAA and **IRN**I + S**G**KAA respectively. The remaining quintuple haplotypes containing the *pfdhfr* triplet mutant **IRN** and different SNP combinations at two *pfdhps* codons (G_43**7**_A_581_) were present in four samples. Only one sample had the *dhfr* triple mutant without any additional mutations.

#### Multilocus haplotypes

Multilocus haplotypes in CQ and SP markers were constructed based on *pfcrt*: K76T, *pfmdr1*: N86Y, *pfdhfr* N_51_I/C_59_R/S_108_N and *dhps* A_437_G/K_540_E to determine if CQ resistant parasites also tend to be SP resistant. In total, seven haplotypes were observed, with 13.7% and 59.8% of samples having variants with 6 (**T + Y + IRN + G**K) and 5 (**T +** N + **IRN + G**K) mutations respectively. Nevertheless, no sample had the sextuple mutant haplotype (**T + Y + IRN + GE**).

## Discussion

Antimalarial drug resistance monitoring remains critical to malaria control and elimination, especially with the confirmation of artemisinin resistance in Cambodia [3, 28] and other foci in that region. ITNs can alter *Plasmodium* spp. transmission and thus indirectly influence the spread of drug resistance by changing the number of parasite clones per host and the level of community/population drug use [6]. The use of ACT is not only expected to improve the treatment efficacy, but also to delay the emergence of *P. falciparum* drug resistance [51]. Therefore, it is very important to monitor ACT partner drugs to ensure that national treatment policies remain effective [52]. In the mount Cameroon area, ITN ownership and usage has increased significantly following the nationwide free distribution campaign [37], possibly selecting for resistant parasites over time. The study assessed molecular markers to a wide panel of antimalarials in this area, across three transects at different altitude and varying malaria transmission intensity based on proxy measure of malaria parasitaemia.

Although artemisinin resistance has not been documented in Africa [11, 49], and *pfkelch13* alleles are probably not under selection [49], monitoring is necessary, as the history of anti-malarial resistance suggests the possibility of it spreading to Africa despite global efforts in its containment. None of the nonsynonymous polymorphisms at N458Y, Y493H, R539T, I543T, R561H and C580Y in the kelch repeat region of K13 propeller domain validated as markers of artemisinin resistance [28, 29] as well as the adjacent A481V and G533C mutations thought to affect the three-dimensional structure of the *K13*-propeller [30] were observed in the surveyed parasite samples as reported previously [10, 53]. Furthermore, even *pfk13*: A578S, the most frequent allele observed in Africa [29, 53], which has recently been reported elsewhere in the country [54] was not detected, although it is not associated with clinical or in vitro resistance to artemisinin [29]. However, the *K13*: K189T mutation (36%, 58/161) was highly prevalent. Other k13 mutant alleles were at such low frequencies, suggesting that further measures are needed, including monitoring at 2–3 time points and investigating sweeps in flanking microsatellites around the DR markers to ascertain that ART resistant parasites are not under evolutionary selection in south western Cameroon.

The *pfcrt*: K76T [15] and *Pfmdr1*: N86Y mutations [20] are thought to be most decisive in CQ resistance, with the latter allele serving to augment CQ resistance in isolates with the former. As such, the *pfcrt*-K76 allele is expected to be selected after almost 15 years of change in national drug policy. The removal of chloroquine drug pressure resulted in the reemergence of chloroquine sensitive parasites in east Africa [55, 56] that reached 100% in Malawi [56] less than 10 years after chloroquine was replaced with SP and dramatically 2 years after introduction of AL [55]. This study reports a high frequency (55.2%) of *pfcrt*: K76T, but low proportion (13.0%) for *pfmdr1*: N86Y relative to previous studies prior to the large scale ITN distribution in Cameroon (*pfcrt*: 76 T: 71–87.1%) versus *pfmdr1*: N86Y (73.8–76%) [42, 44]. The slow decline in the *pfcrt*: K76T mutant since 2004–2009 is in line with previous reports [4] and can be explained by (i) the fixation of the allele in the parasite populations that need more time to recover CQ sensitivity in the absence of CQ pressure or (ii) the high use of amodiaquine (AQ) at the population level that selects for *pfcrt*: K76T alleles. As such, it is not yet possible to reintroduce CQ against *P. falciparum* in the study area. Nevertheless, the decreased prevalence of the *pfmdr1*: 86Y mutation reflects the complete withdrawal of CQ usage in the community [56] but may also be due to its selection by lumefantrine.

In line with previous reports in Cameroon [41, 42, 44], *pfcrt* polymorphisms scanning revealed that the mutant *pfcrt* CV**IET** (Southeast Asian CQ-resistant) haplotype was still the more predominant in the parasite population while the *pfcrt* SVMNT haplotype was not detected in any of the samples analyzed as reported elsewhere [43]. The remaining isolates had the wild type (CQ-sensitive) *pfcrt* CVMNK form, distributed in all three transects in variable frequencies (Table 3). In all, 25.3% (60/237) were of CVMNK type—suggesting that one quarter of *P. falciparum* isolates are still susceptible to chloroquine in the area, slightly higher than previous reports [57]. Such genetic reformation might have been propelled by the selection pressure exerted by the amodiaquine component of the AS-AQ artemisinin combined therapy recommended for the treatment of uncomplicated *P. falciparum* malaria in Cameroon [44]. This can be justified by the fact that AQ has a very similar genetic target (*Pfcrt*) to chloroquine [43]. With close to three quarters of the population carrying this CQR haplotype, however, CQ and AQ cannot be effective treatment options in the area. Taken together, these findings suggest that the intensification of control has not affected the diversity of the parasite population. Nevertheless, that only 47.3% of parasites were of the reversible CQ resistant haplotype (CV**IET**) phenotype suggests the possibility of CQ re-use over time.

Sulphadoxine-pyrimethamine remains the drug of choice by the World Health Organization for intermittent preventive treatment in pregnancy (IPTp) [58], although, resistance is reportedly increasing in stable transmission areas [59, 60]. The *dhfr* IRN triple mutant and *dhps* double GE mutant combination associated with in vivo SP treatment failure [61] was not recorded in any of the samples analyzed. However, up to 99.6% of samples harbored the *dhfr* triple mutant in this study (Table 3) while none of the isolates carried the *dhps*: K540E mutant. This suggests that resistance to pyrimethamine but not sulphadoxine is widespread in the study area, although it may also be due to trimethoprim and sulfamethoxaxole (Cotimoxazole), a commonly used antibiotic that is known to select for *dhfr/dhps* resistant alleles [62]. This suggests that IPTp with SP may no longer be effective in the area, although further measures are needed to confirm the local prevalence of *dhfr/dhps* genotypes/haplotypes. Additionally, the overall impact of these alleles on the IPTp-SP routine can only be ascertained through in vivo efficacy studies in pregnancy.

The *pfdhps*: 142N and *pfmdr1*: 184F mutations were highest at a low altitudes compared to medium and high altitudes. However, there were no significant differences in the prevalence of the critical *pfcrt*: 76T and *pfmdr1*: 86Y mutations as well as CQ *pfcrt* and SP *pfdhfr/dhps* haplotypes among the three transects (Table 3). Although variability in malaria parasitaemia with altitude has been reported [36, 46] in the region, the prevalence of the markers does not mirror this. The similarity in the prevalence of the markers among the three areas could be explained by the small relative differences in transmission intensity between areas as well as gene flow due to migration of human and vector populations [6].

This study had a few limitations. First the small number of samples analysed in this study might have also reduced the statistical power. Secondly, the geographic proximity of the three study areas and evaluation of the effect of transmission intensity on drug resistance at a single time point may have limited the ability to detect differences in the molecular profiles of drug resistance among the areas [6]. Thirdly, the fact that only individuals with asymptomatic parasitaemia or uncomplicated malaria were enrolled may have limited the diversity of the parasite population analysed.

## Conclusions

None of the candidate and validated *K13* resistance mutations were detected in southwestern Cameroon, although other non-synonymous mutations were observed. Parasites in the area, however, remain largely resistant to CQ, with only a slow decline in the *pfcrt*: K76T mutant since 2004–2009 suggesting the fixation of the allele in the populations that need more time to recover CQ sensitivity in the absence of CQ pressure. Resistance to pyrimethamine but not sulphadoxine is also widespread in the study area.

## Additional files

Additional file 1: Multilingual abstracts in the five official working languages of the United Nations. (PDF 805 kb)

Additional file 2: Comparision of alleles across altitudinal zones along the slope of mount Cameroon. (DOCX 12 kb)

## Abbreviations

ACT: Artemisinin-based combination therapies
AL: Artemether lumefantrine
ANOVA: Analyses of variance
AP: asymptomatic parasitaemia
AQ: Amodiaquine
CQ: Chloroquine
dhfr: Dihydrofolate reductase
dhps: Dihydpteroate synthetase
EIR: Entomological inoculation rates
GMPD: Geometric mean parasite density
IPTp: Intermittent preventive treatment of malaria in pregnancy
ITNs: Insecticide-treated nets
K76 T: Lys to Thr at position 76
LM: lumefantrine
MQ: mefloquine
Pfcrt: *Plasmodium falciparum* chloroquine resistance transporter
Pfmdr1: *Plasmodium falciparum* multidrug resistance 1
QN: Quinine
SEA: Southeast Asia
SNPs: Single nucleotide polymorphisms
SP: Sulphadoxine-pyrimethamine
SSA: Sub-Saharan Africa
UM: Uncomplicated Malaria
WWARN: WorldWide Antimalarial Resistance Network
