# Supplementary material for: Molecular markers for artemisinin and partner drug resistance in natural Plasmodium falciparum populations following increased insecticide treated net coverage along the slope of mount Cameroon: cross-sectional study
Source: Infect Dis Poverty. 2017 Nov 6;6:136. doi: 10.1186/s40249-017-0350-y (PMC5674235; doi:10.1186/s40249-017-0350-y)
Supplement: Supplementary file 1 — Multilingual abstracts in the five official working languages of the United Nations. (PDF 805 kb) [file 40249_2017_350_MOESM1_ESM.pdf]

Translation of the abstract into the five official working languages of the United Nations

العلامات الجزيئية لل أرتيميسينين واقترانه بمقاومة العقاقير بين تجمعاتالمتصورة المنجلية الطبيعية بعد زيادة استخدام الناموسيات المعالجة بالمبيدات الحشرية على طول منحدر جبل الكاميرون: دراسة مستعرضة

توبياس و أبينيوه، ريجينا ن موجري، أوليفو ميوتو، هانيش ف تشي، رولاند ب تاتا، جوديث ك أنشانج كيمبي, إليانور م فون، دلفين أ تانجوه، روبرت الخامس نينجشو، كريستوفر جاكوب، روبرتو أماتو، عبد الله دجيمد، دومينيك كوياتكوفسكي، اريك أ أشيدي وألفريد أمبوا-نجوا

ملخص

خلفية: المقاومة للعقاقير واحدة من أكبر التحديات التي تواجه برامج مكافحة الملاريا، مع رصد مقاومة الطفيليات للأرتيميسينين أو للعلاج بتوليفة الأرتيميسينين (ACT) يقترن بشكل حاسم بجهود القضاء على المرض. تم تقييم علامات المقاومة بمجموعة واسعة من الأدوية المضادة للملاريا في المجموعات الطبيعية للطفيلي في جنوب غرب الكاميرون.

الأساليب: التحق الأفراد المصابين بالملاريا غير المصحوبة بمضاعفات أو أعراض الطفيليات بالبحث من خلال استقصاءات مستعرضة في شهر مايو 2013 إلى مارس 2014 على طول منحدر جبل الكاميرون. ع لى ال مح توي ال دم ف حص ت م أعمدة ب ا س تخدام ال ضوئ ي ب ال مجهر ال ب يضاء ال كري ات من م س ت ن فد ال م نجل ية ال م تصورة ملاري ا ال ط ف يل يات HiSeq. إل وم ي نا م نصة ب ا س تخدام ال ط ف يلي ال ج ي ني ل ل نمط ال ت س ل سل من ال تأكد وت م CF11 ال س ل يلوز

النتائج: وقد تم تسجيل 259 مشاركا في هذه الدراسة من ثلاثة ارتفاعات مختلفة. في حين أن بعض الأليلات المرتبطة بمقاومة العقاقير في ، وpfmdr1 وpfcrt كانت شديدة الانتشار، أقل من 3٪ من جميع العينات تحمل الطفرات في جين pfkelch13، ولم يكن أي منها من بين تلك المرتبطة ببطء معدلات تصفية طفيليات الأرتيميسينين في جنوب شرق آسيا. الأنماط الفردانيةpfcrt، pfmdr1، pfdhfr ، pfdhps كانت طفرات ثلاثية لـpfcrt--74 و 75 و 76 أي$_{C72}$الخامس$_{73}$أنا$_{74}$ه$_{75}$ر$_{76}$ (47.3%)، طفرة واحدة في كودون 184، N$_{86}$و$_{184}$د$_{1246}$ (53.2%)، و أنا$_{51}$ص$_{59}$N$_{108}$أنا$_{164}$ (99 ٪) و S$_{436}$G$_{437}$K$_{540}$A$_{581}$A$_{613}$ (69 في المائة) المتغيرات في الجينات على التوالي.

الاستنتاجات: غلبة الطفيليات ثلاثية التحولpfcrt IETCV و dhfr IRN وانعدام مقاومة الآليلات pfkelch13 يوحي بأن مكونات الامودياكومين والبيريميثامين في As-AQ-AS قد لا تكون فعالة في دورها بينما مقاومة الكلوروكين لا تزال موجودة في جنوب غرب الكاميرون.

Translated from English version into Arabic by Mahmoud Sami, through

TRANSLATORS
WITHOUT BORDERS

在喀麦隆山侧面区域增加杀虫剂处理蚊帐后，一项关于恶性疟原虫自然种群对青蒿素和配伍药物抗药性分子标记的横断面研究

Tobias O Apinjoh, Regina N Mugri, Olivo Miotto, Hanesh F Chi, Rolland B Tata, Judith K Anchang-Kimbi, Eleanor M Fon, Delphine A Tangoh, Robert V Nyingchu, Christopher Jacob, Roberto Amato, Abdoulaye Djimde, Dominic Kwiatkowski, Eric A Achidi and Alfred Amambua-Ngwa

摘要

引言：抗药性是疟疾防治规划面临的最大挑战之一。监测疟原虫对青蒿素或青蒿素联合疗法(ACT)的配伍药物的抗药性，对疟疾消除至关重要。本研究对喀麦隆西南部的疟原虫自然种群的一系列抗疟药抗性标记进行了评估。
方法：从2013年5月到2014年3月，在喀麦隆山的侧面区域，将无症状带虫者或无并发症疟疾患者纳入本项横断面调查。利用光学显微镜从血样中筛选出含有恶性疟原虫的血液，采用CF11纤维素柱去除白细胞，通过Illumina HiSeq平台测序以确定疟原虫基因型。
结果：共有来自3个不同海拔地区的259名参与者纳入了本研究。在*pfdhfr*、*pfmdr1*和*pfcrt*中与抗药性相关的一些等位基因非常普遍，所有样本中仅有不到3%的携带pfkelch13基因突变，其中没有一种与东南亚青蒿素疟原虫清除率降低相关。最常见的pfcrt、pfmdr1、pfdhfr和pfdhps单倍型分别为*pfcrt*-74、75和76的三突变体，即$C_{72}V_{73}I_{74}E_{75}T_{76}$(47.3%)，在184位密码子的单碱基突变、$N_{86}\mathbf{F}_{184}D_{1246}$ (53.2%)、 $\mathbf{I}_{51}\mathbf{R}_{59}\mathbf{N}_{108}I_{164}$ (99%)和 $S_{436}\mathbf{G}_{437}K_{540}A_{581}A_{613}$ (69%)。
结论：*pfcrt* **CVIET** 和 *dhfr* **IRN** 三重突变体疟原虫的优势以及 *pfkelch13* 抗性等位基因的缺失表明，在喀麦隆西南部阿莫地喹和 AS-AQ 和 SP 组合的乙胺嘧啶组分可能不再有效，而氯喹抗药性仍然存在。

Translated from English version into Chinese by Xin-Yu Feng, edited by Pin Yang

Marqueurs moléculaires de la résistance à l'artémisinine et aux médicaments associés dans les populations naturelles de *Plasmodium falciparum* après l'extension de la couverture par les moustiquaires imprégnées sur les pentes du mont Cameroun : étude transversale

Tobias O Apinjoh, Regina N Mugri, Olivo Miotto, Hanesh F Chi, Rolland B Tata, Judith K Anchang-Kimbi, Eleanor M Fon, Delphine A Tangoh, Robert V Nyingchu, Christopher Jacob, Roberto Amato, Abdoulaye Djimde, Dominic Kwiatkowski, Eric A Achidi et Alfred Amambua-Ngwa

Résumé
Contexte: La résistance aux médicaments est l'une des plus grandes difficultés auxquelles se heurtent les programmes de lutte contre le paludisme. La surveillance de la résistance du parasite à l'artémisinine ou aux molécules qui lui sont associées dans les combinaisons thérapeutiques à base d'artémisinine (CTA) est cruciale pour l'élimination de la maladie. Les auteurs ont mesuré des marqueurs de résistance à un large panel d'antipaludéens dans des populations naturelles du parasite dans le sud-ouest du Cameroun.
Méthodes: Des sujets présentant une parasitémie asymptomatique ou un paludisme sans complications ont été enrôlés dans des études transversales sur les pentes du mont Cameroun, entre mai 2013 et mars 2014. Le sang présentant une parasitémie à *Plasmodium falciparum* identifiée par microscopie optique a été débarrassé de ses leucocytes sur des colonnes de cellulose CF11 et le génotype du parasite a été déterminé par séquençage sur la plateforme Illumina HiSeq.
Résultats: Au total, 259 participants ont été enrôlés dans cette étude à trois altitudes différentes. Alors que certains allèles associés à la chimiorésistance de *pfdhfr, pfmdr1 et pfcrt* étaient très prévalents, moins de 3 % des échantillons étaient porteurs de mutations du gène *pfkelch13* et aucune de celles-ci

n'était associée à une élimination lente du parasite en Asie du Sud-Est. Les haplotypes les plus prévalents de *pfcrt, pfmdr1, pfdhfr* et *pfdhps* étaient des mutants triples au niveau de *pfcrt*-74, 75 et 76, à savoir $C_{72}V_{73}\mathbf{I_{74}E_{75}T_{76}}$ (47,3 %), une mutation unique sur le codon 184, $N_{86}\mathbf{F}_{184}D_{1246}$ (53,2 %) et les variants $\mathbf{I}_{51}\mathbf{R}_{59}\mathbf{N}_{108}I_{164}$ (99 %) et $S_{436}\mathbf{G}_{437}K_{540}A_{581}A_{613}$ (69 %) des gènes, respectivement.
Conclusions: La prédominance de parasites à triple mutation de *pfcrt* **CVIET** et *dhfr* **IRN** et l'absence d'allèles de résistance de*pfkelch13* suggèrent que l'amodiaquine et la pyriméthamine utilisées dans les traitements ASAQ et SP pourraient ne plus jouer leur rôle, tandis que la résistance à la chloroquine persiste dans le sud-ouest du Cameroun.

Translated from English version into French by Suzanne Assenat, through

TRANSLATORS
WITHOUT BORDERS

Молекулярные маркеры для определения лекарственной устойчивости Plasmodium falciparum в его естественной среде обитания к *артемизинину и его производным,* как следствие увеличения общего охвата территорий, подвергаемых обработке инсектицидами на склонах вулкана Камерун: межгрупповое исследование

Тобиас О Апинджо, Регина Н Мугри, Оливо Миотто, Ханеш Чи Ф, Роллан Б Тата, Джудит К Анчанг-Кимби, Элеанора М Фонь, Дельфин А Танох, Роберт В Ниингчу, Кристофер Якоб, Роберто Амато, Абдулайе Джимде, Доминик Квятковски, Эрик А Ачиди и Альфред Амамбуа-Нгва

Аннотация
Справочная информация: Устойчивость к лекарственным препаратам представляет собой одну из наиболее значительных проблем в рамках программ по борьбе с малярией, ключевым элементом в работе по ликвидации которой является мониторинг устойчивости паразита к артемизинину, а также к производным лекарствам комбинированной терапии (ACT), созданным на его основе. Оценка маркеров устойчивости к широкому спектру противомалярийных препаратов производилась в естественной среде обитания паразитов на юго-западе Камеруна.
Методы: В период с мая 2013 года по март 2014 год в межгрупповые обследования были включены лица с бессимптомной паразитемией, а также неосложненной малярией, проживающие на территориях вдоль склона вулкана Камерун. *Plasmodium falciparum* Пониженный уровень лейкоцитов был установлен путём секвенирования методом Illumina HiSeq при анализе заражённой малярией крови под световым микроскопом с использованием целлюлозной хроматографической бумаги CF11 и генотипа паразитов.
Результаты: В исследовании приняло участие в общей сложности 259 пациентов, проживающих на трёх различных высотах. Хотя в устойчивости к лекарственным препаратам генов *pfdhfr, pfmdr1 and pfcrt* существенно превалировали некоторые аллели, мутации наблюдались в менее 3% всех образцов гена *pfkelch13* , ни один из которых не был связан с

медленными темпами очистки Юго-Восточной Азии от данного паразита при помощи артемизинина. Наиболее распространенные гаплотипы *pfcrt, pfmdr1, pfdhfr* и гаплотипы *pfdhps* оказались тройными мутантами *pfcrt*-74, 75 и 76, т.е. $C_{72}V_{73}\mathbf{I}_{74}\mathbf{E}_{75}\mathbf{T}_{76}$ (47,3%), единственная мутация в кодоне 184, $N_{86}\mathbf{F}_{184}D_{1246}$ (53,2%), $\mathbf{I}_{51}\mathbf{R}_{59}\mathbf{N}_{108}I_{164}$ (99%), а также $S_{436}\mathbf{G}_{437}K_{540}A_{581}A_{613}$ (69%) разновидностей в соответствующих генах.
Выводы: Преобладание тройных мутантов-паразитов *pfcrt* CV**IET** **и** *dhfr* **IRN**, а также отсутствие устойчивых аллелей *pfkelch13* позволяет предположить, что компоненты амодиакина и пириметамина в AS-AQ и SP более не могут быть эффективными в выполнении своих функций, в то время как устойчивость к хлорохину до сих пор сохраняется на юго-западе Камеруна.

Translated from English version into Russian by Liudmila Tomanek (nee Volynets), through

Marcadores moleculares para la resistencia a la artemisinina y su fármaco asociado en poblaciones de *Plasmodium falciparum* a raíz del aumento de la cobertura neta tratada con insecticida en la ladera del monte Camerún: Estudio transversal

Tobias O Apinjoh, Regina N Mugri, Olivo Miotto, F de Hanesh, Rolland B Tata, Judith K Anchang-Kimbi, Eleanor M Fon, Delphine A Tangoh, Robert V Nyingchu, Christopher Jacob, Roberto Amato, Abdoulaye Djimde, Dominic Kwiatkowski, Eric A Achidi y Alfred Amambua-Ngwa

Resumen
Contexto: La resistencia a los fármacos es uno de los mayores retos para los programas de control de la malaria y la vigilancia de la resistencia del parásito a la artemisinina o a la terapia combinada de artemisinina y otros fármacos es fundamental para los esfuerzos que tienen como objetivo su eliminación. Se evaluaron los marcadores de resistencia a un amplio conjunto de fármacos antimaláricos en las poblaciones de parásitos naturales del suroeste de Camerún.
Métodos: Se inscribió a individuos con parasitemia asintomática o malaria sin complicaciones mediante estudios transversales entre mayo de 2013 y marzo de 2014 en la ladera del Monte Camerún. En la sangre de malaria parasitaria con *Plasmodium falciparum* examinada con el microscopio se eliminaron los leucocitos mediante columnas de celulosa CF11, así como el genotipo del parásito determinado por la secuencia de la plataforma Illumina HiSeq.
Resultados: En total se inscribieron 259 participantes en este estudio a tres altitudes diferentes. Si bien algunos alelos asociados con la resistencia a los fármacos en *pfdhfr y pfmdr1 pfcrt* eran muy frecuentes, menos del 3% de todas las muestras experimentaron mutaciones en el gen *pfkelch13* , ninguno de los cuales estaban asociados a las bajas tasas de eliminación del parásito artemisinina en el sudeste asiático. Los haplotipos prevalentes de *pfcrt pfmdr1, pfdhfr* y *pfdhps* fueron triples mutantes de *pfcrt* -74, 75 y

76, es decir $C_{72}V_{73}\mathbf{I_{74}E_{75}T_{76}}$ (47.3%), una sola mutación en el codón 184, $N_{86}\mathbf{F}_{184}D_{1246}$ (53,2%), el $_{51}\mathbf{R}_{59}\mathbf{N}_{108}I_{164}$ (99%) y $S_{436}\mathbf{G}_{437}K_{540}A_{581}A_{613}$ (69%) las variantes en los genes, respectivamente.
Conclusiones: El predominio de los parásitos con triple mutación de *pfcrt* CV**IET y** *dhfr* **IRN** y la ausencia de alelos de resistencia *pfkelch13* sugieren que los componentes de AS-AQ y SP amodiaquina y pirimetamina ya no pueden ser eficaces en su papel, mientras que la resistencia a la cloroquina persiste en el suroeste de Camerún.

Translated from English version into Spanish by SergioLorenzi, through

TRANSLATORS
WITHOUT BORDERS
