# Supplementary material for: Molecular markers for artemisinin and partner drug resistance in natural Plasmodium falciparum populations following increased insecticide treated net coverage along the slope of mount Cameroon: cross-sectional study
Source: Infect Dis Poverty. 2017 Nov 6;6:136. doi: 10.1186/s40249-017-0350-y (PMC5674235; doi:10.1186/s40249-017-0350-y)
Supplement: Supplementary file 2 — Comparision of alleles across altitudinal zones along the slope of mount Cameroon. (DOCX 12 kb) [file 40249_2017_350_MOESM2_ESM.docx]

Supplementary Tables

Table S1

| **Gene** | **Codons** | **All samples** | **MT** |  |  |  |
| --- | --- | --- | --- | --- | --- | --- |
|  |  |  |  | **MM** | **CB** | ***P*-value** |
|  | C72S |  |  |  |  |  |
| CRT | M74I | 116 (55.0) | 16 (64.0) | 71 (54.2) | 26 (54.2) | 0.652 |
|  | N75E | 115 (54.2) | 16 (64.0) | 70 (53.4) | 26 (53.1) | 0.604 |
|  | K76T(R,I) | 116 (55.2) | 16 (64.0) | 71 (54.6) | 26 (54.2) | 0.668 |
|  | Q271K | 120 (62.5) | 17 (68.0) | 74 (61.7) | 26 (65.0) | 0.809 |
|  | I356K | 77 (46.1) | 7 (41.2) | 48 (45.3) | 19 (50.0) | 0.819 |
| DHFR | N51I | 232 (99.6) | 28 (100) | 144 (99.3) | 54 (100) | 0.755 |
|  | C59R | 232 (99.6) | 28 (100) | 144 (99.3) | 54 (100) | 0.755 |
| DHPS | K142N | 19 (9.2) | 6 (26.1) | 8 (6.2) | 3 (6.1) | **0.006** |
|  | I431V | 31 (17.6) | 2 (8.7) | 24 (21.8) | 5 (13.2) | 0.221 |
|  | S436A | 39 (32.0) | 3 (30.0) | 30 (36.6) | 6 (25.0) | 0.554 |
|  | A581G | 19 (9.8) | 1 (4.2) | 17 (14.0) | 1 (2.4) | 0.061 |
|  | A613S | 18 (12.3) | 2 (12.5) | 15 (16.3) | 1 (3.1) | 0.159 |
| MDR1 | N86Y | 25 (13.0) | 4 (17.4) | 17 (14.0) | 4 (9.3) | 0.611 |
|  | Y184F | 121 (72.0) | 17 (94.4) | 77 (73.3) | 23 (56.4) | **0.010** |
| KELCH13 | K189T | 58 (36.0) | 7 (36.8) | 31 (31.3) | 18 (47.4) | 0.214 |
